# Mitochondrial Genomic Variation in Rare and Metabolic Diseases: From Heteroplasmy to Precision Medicine

**DOI:** 10.3390/life16101590

**Published:** 2026-09-23

**Authors:** Arijahir Alexis Mancio-Cárdenas, Humberto García-Ortiz, Francisco Barajas-Olmos, Cecilia Contreras-Cubas, Ximena Luna-González, Diego Ramírez-Martínez, Federico Centeno-Cruz, José Rafael Villafán-Bernal, Montserrat Ivonne Morales-Rivera, Beth Mitchell Gamez-Rangel, Angélica González-Oliver, Lorena Orozco, Angélica Martínez-Hernández

**Affiliations:** 1 Posgrado en Ciencias Biológicas, Facultad de Medicina, Universidad Nacional Autónoma de México, Mexico City 04510, Mexico; alexismancio@hotmail.com (A.A.M.-C.); dmartinez@inmegen.edu.mx (D.R.-M.); biologabethrangel@gmail.com (B.M.G.-R.); 2 Immunogenomics and Metabolic Diseases Laboratory, Instituto Nacional de Medicina Genómica, Secretariat of Health, Mexico City 14610, Mexico; hgarcia@inmegen.gob.mx (H.G.-O.); fbarajas@inmegen.gob.mx (F.B.-O.); ccontreras@inmegen.gob.mx (C.C.-C.); ximenalunaglez01@gmail.com (X.L.-G.); fcenteno@inmegen.gob.mx (F.C.-C.); joravibe@hotmail.com (J.R.V.-B.); mrivera@inmegen.edu.mx (M.I.M.-R.); 3 Investigador por México, Secretaría de Ciencia, Humanidades, Tecnología e Innovación (SECIHTI), Mexico City 03940, Mexico; 4 Departamento de Biología Celular, Facultad de Ciencias, Universidad Nacional Autónoma de México, Mexico City 04510, Mexico; goliver@ciencias.unam.mx

**Keywords:** mitochondrial disease, heteroplasmy, metabolic disorders, next-generation sequencing, oxidative phosphorylation, bioenergetics

## Abstract

Mitochondrial DNA (mtDNA) variation has traditionally been investigated in the context of human evolution and rare mitochondrial diseases; however, growing evidence suggests that it may also contribute to susceptibility to common metabolic disorders. This review integrates current knowledge of mitochondrial biology, mtDNA genetics, heteroplasmy, and the global distribution of mitochondrial haplogroups to examine their roles in health and disease. In this narrative review, we synthesized English-language studies identified through PubMed, Scopus, and Web of Science from January 1988 to May 2026, prioritizing evidence according to relevance, scientific contribution, recency, and historical significance. We summarize evidence showing how pathogenic variants (PVs) in mtDNA genes impair oxidative phosphorylation (OXPHOS), increase reactive oxygen species (ROS) production, and disrupt cellular bioenergetics, thereby contributing to the multisystem manifestations of mitochondrial diseases. We further discuss population-specific mitochondrial haplogroups and their reported population-specific associations with metabolic phenotypes, including obesity, type 2 diabetes (T2D), and cardiovascular disease (CVD). Finally, we review recent advances in next-generation sequencing technologies and their contributions to the molecular diagnosis of mitochondrial diseases and the characterization of genotype–phenotype relationships. Collectively, current evidence supports a well-established pathogenic role for specific mtDNA variants in primary mitochondrial disorders, whereas the contributions of common polymorphisms and haplogroups to complex metabolic disease remain largely associative and require further replication and functional validation.

## 1. Introduction

Mitochondrial DNA (mtDNA) variants have been fundamental to reconstructing maternal ancestry, defining migratory routes, and elucidating genetic relationships among human populations. Beyond their evolutionary and anthropological relevance, mtDNA variants are also of biomedical interest because the mitochondrial genetic background can influence cellular bioenergetics and may contribute to differences in metabolic phenotypes and disease susceptibility [1,2].

Mitochondrial diseases (MitDs) comprise a clinically and genetically heterogeneous group of disorders caused by defects in the oxidative phosphorylation system (OXPHOS) and other aspects of mitochondrial function. These conditions arise from pathogenic variants (PVs) in either the mitochondrial genome or in nuclear genes encoding mitochondrial proteins and other factors required for mitochondrial homeostasis [1,3].

At the cellular level, PVs can impair ATP synthesis and increase reactive oxygen species (ROS) production. These alterations may initiate a self-perpetuating cycle of oxidative and metabolic stress that promotes mitochondrial dysfunction and disease progression. mtDNA PVs are directly implicated in primary mitochondrial disorders, whereas common mtDNA variants and haplogroups have primarily been investigated in relation to population history and their associations with complex phenotypes. Emerging evidence also implicates mitochondrial dysfunction in the development of common metabolic disorders [3,4], although these associations must be interpreted in the context of population structure, demographic history, environmental factors, and potential confounding by ancestry [5].

Mitochondrial genomic studies in populations with substantial Native American ancestry remain limited, restricting the characterization of population-specific mitochondrial genetic diversity and demographic history in Latin America [6]. This gap may also limit our understanding of the contribution of mtDNA variation to disease susceptibility in these populations.

This review integrates evidence on mitochondrial genomic variation across the spectrum from primary mitochondrial disorders to secondary mitochondrial dysfunction and common complex metabolic diseases. To provide a clear conceptual framework, we distinguish five biologically and clinically distinct categories: (1) inherited pathogenic mtDNA variants and heteroplasmy in primary mitochondrial disorders; (2) common inherited mtDNA polymorphisms and mitochondrial haplogroups, which primarily reflect maternal population history and have been investigated as modifiers of susceptibility to complex traits; (3) somatic mtDNA alterations that accumulate during ageing and cellular stress and may contribute to mitochondrial genomic instability; (4) mtDNA copy-number changes, representing a quantitative mitochondrial genomic feature whose interpretation is strongly influenced by tissue and analytical methodology; and (5) general or secondary mitochondrial dysfunction occurring in metabolic, inflammatory, toxic, neurodegenerative, and other acquired conditions without necessarily representing a primary inherited mitochondrial disease. We also examine advances in genomic technologies, particularly next-generation sequencing (NGS), for the detection, characterization, and interpretation of these different forms of mitochondrial genomic variation. Particular emphasis is placed on how mitochondrial population history and the limited representation of Latin American and Native American-ancestry populations may affect the interpretation of mitochondrial genetic variation and disease susceptibility.

Unlike reviews focused primarily on inherited mitochondrial diseases or on isolated associations between mtDNA haplogroups and metabolic traits, this review brings these complementary areas together within a population-aware genomic framework. By connecting primary mitochondrial disorders, secondary mitochondrial dysfunction, common mtDNA diversity, and mitochondrial population history, we highlight both established clinical applications and the current limitations of mitochondrial genomics in precision medicine.

## 2. Literature Search and Evidence Selection

A literature search was conducted in PubMed, Scopus, and Web of Science to identify relevant literature from January 1988 to May 2026. The search included combinations of the following terms: mtDNA, MitDs, heteroplasmy, mitochondrial haplogroup, OXPHOS, mitochondrial dysfunction, obesity, T2D, CVD, next-generation sequencing (NGS), whole-genome sequencing (WGS), whole-exome sequencing (WES), and precision medicine, using Boolean operators as appropriate. We considered English-language original research studies, including observational, genomic, and population-genetic investigations, as well as relevant systematic reviews and case reports. Evidence was selected using a narrative, relevance-based approach that considered thematic relevance, methodological contribution, recency, population representation, and historical importance. Recent studies were prioritized for contemporary clinical and genomic topics. For mitochondrial haplogroup associations with metabolic diseases, we included studies conducted in different populations, including reports of positive, null, or conflicting associations, to provide a balanced representation of the available evidence. Because this article was designed as a narrative synthesis rather than a systematic review, no formal systematic-review protocol, meta-analysis, or standardized risk-of-bias assessment was performed.

## 3. Mitochondria: Structure, Function, and Genetics

### 3.1. Mitochondrial Genome Organization

Mitochondria are widely recognized as the “powerhouses of the cell”; however, their biological functions extend far beyond ATP production. They play essential roles in metabolic regulation, ROS generation and detoxification, ionic homeostasis, intracellular signaling, apoptosis, and the coordination of multiple stress-response pathways essential for cellular homeostasis [5,7]. Given these diverse functions, mitochondrial integrity is critical for maintaining cellular and systemic physiological balance.

Structurally, mitochondria are double-membrane organelles with distinct lipid compositions and specialized protein systems. The outer mitochondrial membrane (OMM) acts as a selectively permeable barrier, allowing the diffusion of small molecules such as ions and small metabolites through voltage-dependent anion channels, also known as porins. In addition to its role in metabolic exchange, the OMM is a key regulator of apoptosis, controlling the release of pro-apoptotic proteins, such as cytochrome c, into the cytosol. It also participates in the import of nuclear-encoded mitochondrial proteins synthesized in the cytosol through specialized translocase complexes, including the translocase of the outer membrane (TOM) complex [8,9]. In contrast, the inner mitochondrial membrane (IMM) is highly folded into structures known as cristae, which markedly increase membrane surface area and optimize the efficiency of oxidative phosphorylation. The IMM is rich in proteins and specialized lipids and is highly impermeable to ions, a property essential for maintaining the proton gradient that drives ATP synthesis via oxidative phosphorylation. The IMM houses the multiprotein complexes of the electron transport chain (Complexes I–IV) and ATP synthase (Complex V), which together drive ATP synthesis. Moreover, the IMM plays a crucial role in maintaining the mitochondrial membrane potential, regulating ROS production, and coordinating mitochondrial dynamics and bioenergetic adaptation in response to cellular demands [9].

The intermembrane space (IMS) contains proteins involved in mitochondrial metabolism and signaling, including cytochrome c and enzymes such as adenylate kinase. During oxidative phosphorylation, protons (H^+^) are pumped from the mitochondrial matrix into the IMS, contributing to the electrochemical gradient required for ATP synthesis [8,9].

The mitochondrial matrix (MM) contains mtDNA and harbors numerous enzymes involved in mitochondrial metabolism, including those catalyzing the first two reactions of the urea cycle, carbamoyl phosphate synthetase 1 (CPS1) and ornithine transcarbamylase (OTC), whereas the remaining reactions occur in the cytosol [9].

Human mtDNA is a circular, intronless, double-stranded DNA molecule comprising 16,569 base pairs and encoding 37 genes: 13 OXPHOS subunits, 2 ribosomal RNAs (rRNAs), and 22 transfer RNAs (tRNAs). The genome consists of a heavy (H) strand, enriched in purines and encoding 28 genes, and a light (L) strand, enriched in pyrimidines and encoding the remaining 9 genes [9]. mtDNA is organized into protein-DNA complexes known as nucleoids, in which mitochondrial transcription factor A (TFAM) is a major structural component that contributes to mtDNA packaging and compaction. Nucleoids also contain proteins involved in mtDNA replication, transcription, and maintenance, and variation in the TFAM-to-mtDNA ratio can influence nucleoid compaction and transcriptional and replicative activity [10].

A critical non-coding region of mtDNA, the displacement loop (D-loop), spans approximately nucleotide positions 16,024-576 and contains the major regulatory elements for mitochondrial transcription and the origin of the H-strand (OH). In contrast, the origin of L-strand replication (OL) is located outside the D-loop [9,11]. The D-loop contains multiple mutational hotspots that define three hypervariable regions: the hypervariable region I (HV1, nucleotides 16,024–16,365); hypervariable region II (HV2, nucleotides 73–340); and hypervariable region III (HV3, nucleotides 438–576). These regions are widely used in population genetics and phylogenetic studies because of their high sequence variability [12,13,14]. mtDNA is characterized by a distinctive mutational landscape influenced by replication dynamics and exposure to oxidative and other forms of cellular stress. Nevertheless, mitochondria possess DNA repair mechanisms, particularly base excision-repair, that contribute to maintaining mitochondrial genome integrity [4,5,15].

Finally, most mitochondrial proteins are encoded by nuclear DNA, synthesized on cytosolic ribosomes, and subsequently imported into mitochondria [8,9]. These proteins support diverse mitochondrial processes, including OXPHOS, mtDNA replication, transcription and translation, mitochondrial dynamics, protein import, metabolite transport, iron-sulfur cluster assembly, and metabolic pathways [16]. Consequently, mitochondrial function depends on coordinated communication between the mitochondrial and nuclear genomes. This mito-nuclear interaction is particularly relevant to OXPHOS because Complexes I, III, IV, and V contain subunits encoded by both genomes and therefore require tightly coordinated expression, import, and assembly. Disruption of this coordination can compromise respiratory-chain function, ATP production, redox homeostasis, and cellular metabolism [16]. Moreover, mitochondria communicate their functional and metabolic status to the nucleus through retrograde signaling pathways that can influence nuclear gene expression in response to metabolic and cellular stress [16,17]. Therefore, mitochondrial disease should be considered within the broader context of mito-nuclear interactions rather than solely as a consequence of mtDNA abnormalities.

### 3.2. Heteroplasmy and the Threshold Effect

Heteroplasmy refers to the coexistence of different mtDNA genotypes within the same cell, tissue, or individual. Importantly, heteroplasmy is dynamic; its level may vary among tissues within the same individual and can change throughout life, contributing substantially to the clinical variability observed in mitochondrial disorders [18,19]. The distribution of heteroplasmy is influenced by several biological processes. During oogenesis and early embryonic development, the mitochondrial genetic bottleneck can produce marked shifts in the proportion of mtDNA variants transmitted from a mother to her offspring [19,20]. Consequently, individuals within the same maternal lineage may inherit different heteroplasmy levels and therefore exhibit different clinical manifestations. During embryonic development, replicative segregation can further alter heteroplasmy levels through the random distribution of mtDNA molecules during cell division [20]. In addition, clonal expansion of specific mtDNA variants and tissue-specific selective pressures can contribute to the progressive enrichment or depletion of particular mitochondrial genomes over time. These processes help explain why heteroplasmy can differ considerably across tissues and change with ageing [19].

The tissue distribution of heteroplasmy has important clinical implications. mtDNA PVs may be detected at different levels across tissues, and heteroplasmy measured in blood may not accurately reflect the mutational burden in clinically affected tissues, particularly for variants undergoing age-related or tissue-specific selection [21,22]. Therefore, interpretation of mtDNA variants requires consideration of the tissue analyzed, the patient’s age and phenotype, maternal inheritance, and the biological characteristics of the specific variant. When clinical suspicion remains high despite a negative or low-level result in blood, analysis of an alternative or clinically affected tissue may provide greater diagnostic sensitivity [21].

The phenotypic consequences of heteroplasmy are closely related to the concept of the threshold effect. Importantly, there is no universal heteroplasmy threshold that reliably predicts biochemical or clinical dysfunction across all mtDNA variants, tissues, or disease phenotypes. Instead, the relationship between heteroplasmy and functional impairment is highly context-dependent and may vary according to the specific mtDNA variant, affected gene, tissue and cell type, mtDNA copy number, cellular energetic requirements, age, and nuclear genetic background [21,22,23]. Consequently, substantial differences in functional impact may occur at similar heteroplasmy levels across tissues or among individuals carrying the same variant. Heteroplasmy should therefore be interpreted in a variant-, tissue-, and phenotype-specific context rather than according to a single numerical threshold [21,22,23]. Understanding the dynamic and tissue-specific nature of heteroplasmy is therefore essential not only for elucidating disease mechanisms and genotype–phenotype relationships, but also for improving molecular diagnosis and genetic counseling [21].

## 4. Genetics of Mitochondrial Diseases

Mitochondrial diseases comprise a clinically and genetically heterogeneous group of disorders caused by impaired mitochondrial function, particularly OXPHOS, resulting from PVs in either mtDNA or nuclear DNA. Their manifestations range from tissue-specific phenotypes to severe multisystem disorders, reflecting the widespread dependence of tissues on mitochondrial energy metabolism. Primary MitDs are directly caused by such genetic defects, whereas secondary mitochondrial dysfunction can occur in diverse genetic, metabolic, toxic, and neurodegenerative conditions and should not necessarily be considered an inherited mitochondrial disease [24].

### 4.1. Primary Mitochondrial Disorders

Although primary MitDs are individually rare, prevalence estimates vary according to age at onset, diagnostic criteria, and the populations studied. Childhood-onset MitDs occur at an estimated frequency of 5–15 cases per 100,000 individuals [25], whereas adult-onset forms have an estimated prevalence of 9.6 cases per 100,000 individuals for mtDNA-related disorders and 2.9 cases per 100,000 individuals for nuclear DNA-related disorders [26]. These disorders are frequently progressive and are associated with substantial morbidity and mortality. Their marked genetic and phenotypic heterogeneity, multisystem involvement, and variable age at onset can complicate diagnosis and contribute to prolonged diagnostic journeys.

In mtDNA-related disorders, assessment of heteroplasmy is particularly important because the heteroplasmy level and tissue distribution of PVs may influence their functional consequences and clinical expression [19,21]. The diagnostic landscape has changed substantially with the development of NGS technologies, which enable comprehensive analysis of nuclear genes when adequate sequencing coverage and validated analytical pipelines are available. Molecular diagnosis should also account for tissue-specific differences in heteroplasmy to improve diagnostic sensitivity and genotype–phenotype interpretation [21].

Mitochondrial genomic studies in Latin American populations remain limited, restricting the characterization of population-specific mtDNA diversity and its potential clinical relevance [6]. Greater representation of Latin American populations in mitochondrial genomic studies is therefore needed to improve variant interpretation and characterize disease-associated mitochondrial variation.

To illustrate the genetic and clinical heterogeneity of primary MitDs, five representative disorders were selected based on their distinct molecular mechanisms, inheritance patterns, tissue involvement, and clinical presentations: Leber hereditary optic neuropathy (LHON), mitochondrial encephalomyopathy with lactic acidosis and stroke-like episodes (MELAS), Leigh syndrome (LS), myoclonic epilepsy with ragged-red fibers (MERRF), and Kearns–Sayre syndrome (KSS) [27,28]. Collectively, these disorders encompass different forms of mitochondrial genetic variation, including single-nucleotide variants in mtDNA and nuclear genes, variants affecting mitochondrial tRNAs, and large-scale mtDNA rearrangements. These genetic defects can disrupt OXPHOS and other processes essential for mitochondrial energy metabolism, resulting in tissue-specific or multisystem manifestations. Clinical expression is further influenced by the underlying genetic defect, heteroplasmy level and tissue distribution, as well as by mitochondrial and nuclear genetic backgrounds, resulting in complex genotype–phenotype relationships. For each condition, we summarize the major PVs or genes, inheritance patterns, the role of heteroplasmy when applicable, genotype–phenotype correlations, diagnostic strategies, and current therapeutic approaches (Table 1).

#### 4.1.1. Leber Hereditary Optic Neuropathy (LHON)

LHON (OMIM #535000) is one of the most common primary MitDs, and predominantly affects young adult males. Prevalence estimates vary among populations, ranging from approximately 1 in 27,000 to 1 in 54,000 among individuals of European ancestry [29,30]. LHON is characterized by painless, subacute bilateral central vision loss caused by selective degeneration of retinal ganglion cells and subsequent optic nerve atrophy. Visual impairment typically begins between the second and fourth decades of life, although onset may occur from childhood to late adulthood [30]. Approximately 90–95% of LHON cases are caused by three mtDNA PVs: m.11778G>A in *MT-ND4*, m.3460G>A in *MT-ND1*, and m.14484T>C in *MT-ND6*. Among these, m.11778G>A is the most prevalent, accounting for approximately 70% of cases [31]. LHON-associated variants are frequently homoplasmic, although heteroplasmy may also occur. Disease expression is characterized by incomplete penetrance and variable clinical expressivity [30].

Diagnosis is based on the characteristic clinical and ophthalmological phenotype together with molecular confirmation of a pathogenic mtDNA variant. Targeted testing for the three primary LHON-associated variants is commonly performed first, followed by broader mtDNA sequencing when targeted analysis is negative and clinical suspicion remains high [32]. Current therapeutic approaches aim to reduce oxidative stress and improve mitochondrial function, with greater benefit reported when treatment is initiated early in the disease course. Supportive management includes visual rehabilitation and avoidance of potential mitochondrial toxins, particularly smoking and excessive alcohol consumption [32].

#### 4.1.2. Mitochondrial Encephalomyopathy, Lactic Acidosis, and Stroke-like Episodes (MELAS)

MELAS syndrome (OMIM #540000) typically manifests during childhood or early adulthood and has an estimated prevalence of 1 in 10,000 individuals [33,34]. MELAS is characterized by substantial clinical heterogeneity, including stroke-like episodes, seizures, encephalopathy, lactic acidosis, myopathy, hearing loss, diabetes, short stature, and exercise intolerance. Additional neurological features, such as ataxia, peripheral neuropathy, dementia, ophthalmoplegia, and pigmentary retinopathy, may also occur, together with multisystem involvement affecting the cardiovascular, endocrine, and renal systems. Recurrent stroke-like episodes are the clinical hallmark of MELAS and represent a major cause of disability and premature mortality [35]. Most cases are associated with PVs in *MT-TL1*, particularly m.3243A>G, which accounts for approximately 80% of affected individuals, followed by the less frequent m.3271T>C variant. These variants impair mitochondrial tRNA-Leu function and consequently alter mitochondrial protein synthesis, OXPHOS, and ATP production [35].

The diagnosis of MELAS can be challenging because of its substantial clinical variability and the tissue-specific distribution of mtDNA heteroplasmy. Molecular evaluation should therefore account for tissue-specific differences in heteroplasmy [35]. Current management is multidisciplinary and primarily supportive, focusing on the treatment of seizures, stroke-like episodes, and associated systemic complications, including metabolic and cardiac manifestations. L-arginine and L-citrulline have been used in some clinical settings; however, their efficacy remains uncertain and requires validation in well-designed clinical trials [36,37,38].

#### 4.1.3. Leigh Syndrome (LS)

LS (OMIM #256000) is a severe neurodegenerative mitochondrial disorder that affects approximately 1 in 40,000 births [39]. Onset typically occurs in infancy or early childhood and is characterized by developmental delay or regression, hypotonia, muscle weakness, seizures, respiratory dysfunction, and lactic acidosis. Additional neurological and multisystem manifestations may occur, reflecting the broad genetic and phenotypic heterogeneity of the disease. LS is frequently progressive and is associated with substantial morbidity and premature mortality [39].

LS is genetically heterogeneous, involving more than 100 nuclear genes and 16 mtDNA genes. Commonly reported mtDNA variants included m.8993T>G/C, m.9176T>G, and m.9185T>C in *MT-ATP6*; m.13513G>A in *MT-ND5;* m.10197G>A, m.10191T>C and m.10158T>C in *MT-ND3;* and m.3697G>A in the *MT-ND1* gene (also linked to MELAS) [39,40]. This broad genetic spectrum contributes to substantial phenotypic variability and complicates molecular diagnosis.

The inheritance pattern of LS depends on the underlying genetic defect and may be mitochondrial, autosomal recessive, X-linked, or, less frequently, autosomal dominant. Diagnosis integrates clinical and biochemical findings, characteristic bilateral and symmetric lesions involving the basal ganglia and/or brainstem on neuroimaging, and molecular genetic testing [41,42].

#### 4.1.4. Myoclonic Epilepsy and Ragged-Red Fibers (MERRF)

MERRF (OMIM #545000) is a multisystem mitochondrial disorder with an estimated prevalence of 1 per 100,000 individuals and predominantly affects children and young adults. Clinically, the disorder is classically characterized by the combination of myoclonus, seizures, generalized epilepsy, cerebellar ataxia, muscle weakness, and the presence of ragged-red fibers (RRFs) on muscle biopsy. Other manifestations include myopathy, cardiac arrhythmias, hearing loss, optic atrophy, and cognitive decline [43,44].

MERRF-associated mtDNA variants are typically heteroplasmic, and their levels may vary among tissues and individuals. To date, more than 30 mtDNA PVs have been associated with MERRF. Variants in *MT-TK,* which encodes tRNA-Lys, are the predominant molecular cause of MERRF (approximately 90%), with m.8344A>G detected in more than 80% of individuals with the typical clinical phenotype. Other mitochondrial tRNA genes harboring PVs linked to MERRF include *MT-TF*, *MT-TH*, *MT-TI*, *MT-TL1*, *MT-TP*, *MT-TS1*, and *MT-TS2* [43,44,45].

Diagnosis is based on the integration of clinical and molecular findings, with molecular confirmation of mtDNA PVs being central to establishing the diagnosis. Biochemical findings, such as elevated lactate levels in blood or cerebrospinal fluid, and characteristic histopathological findings, including RRFs on muscle biopsy, may provide complementary diagnostic evidence [43,44].

There is currently no established disease-modifying therapy for MERRF. Management is therefore symptomatic and multidisciplinary and may include treatment of seizures and myoclonus, cardiac surveillance, audiological assessment, physical rehabilitation, and management of other neurological and systemic complications [30,45].

#### 4.1.5. Kearns–Sayre Syndrome (KSS)

KSS (OMIM #530000) is a multisystem mitochondrial disorder with an estimated prevalence of 1–3 per 100,000 individuals. The disease is characterized by impaired oxidative metabolism and reduced ATP synthesis, with symptom onset typically before 20 years of age. The classic triad includes chronic progressive external ophthalmoplegia, pigmentary retinopathy, and cardiac conduction defects, often accompanied by ptosis, cerebellar ataxia, hearing loss, short stature, and endocrine dysfunction, such as diabetes and hypothyroidism [46,47].

At the molecular level, KSS is primarily associated with large-scale mtDNA rearrangements rather than point mutations. The most common alteration is a 4977-bp deletion, although multiple deletions and duplications have been reported, including m.8470_13446del, m.8350_13450del, and m.10759_14602del, among others [48]. These deletions are typically heteroplasmic and arise sporadically, with maternal transmission being uncommon. The heteroplasmy level and tissue distribution of deleted mtDNA molecules contribute to clinical variability [49]. Diagnosis is based on the characteristic clinical phenotype together with molecular detection of a large-scale mtDNA deletion.

Because the proportion of deleted mtDNA may vary among tissues, analysis of an affected tissue, particularly skeletal muscle, may be required when blood testing is negative despite strong clinical suspicion [49]. Currently, no established disease-modifying therapy is available; therefore, management is primarily supportive and multidisciplinary, with particular emphasis on cardiac surveillance and the management of conduction abnormalities [49].

### 4.2. Secondary Mitochondrial Dysfunction in Complex Metabolic Disorders

Beyond primary mitochondrial disorders, mitochondrial dysfunction has increasingly been implicated in the pathogenesis of common complex diseases. Metabolic disorders such as T2D, obesity, and metabolic syndrome represent prominent examples in which impaired mitochondrial homeostasis may contribute to disease development and progression [4,50,51].

Genetic or functional alterations affecting OXPHOS components may impair respiratory-chain assembly and function, leading to increased electron leakage, reduced proton pumping across the IMM, and decreased ATP production. The extent of mitochondrial dysfunction may also be influenced by environmental and metabolic factors, including dietary patterns, physical activity, and other metabolic stressors. Impaired mitochondrial bioenergetics and persistent oxidative stress can disrupt key metabolic pathways, including oxidative phosphorylation, the tricarboxylic acid cycle, fatty acid β-oxidation, and ketogenesis, potentially contributing to lipid accumulation, impaired glucose utilization, altered insulin signaling, dyslipidemia, chronic inflammation, and vascular dysfunction. Importantly, the relationship between mitochondrial dysfunction and metabolic disease is bidirectional: mitochondrial abnormalities may contribute to metabolic impairment, whereas nutrient overload, insulin resistance, inflammation, oxidative stress, and ageing-related processes can, in turn, further compromise mitochondrial function and mtDNA integrity (Figure 1) [52,53]. In this context, it is important to distinguish inherited mtDNA PVs with established causal roles, including those underlying monogenic mitochondrial diabetes, from other forms of mitochondrial genomic variation investigated in complex metabolic diseases.

In contrast to inherited germline mtDNA variants, somatic mtDNA alterations are acquired during life and are not transmitted through the maternal lineage. These alterations may accumulate in different tissues with ageing and contribute to mitochondrial genomic instability and impaired mitochondrial function [18,54]. Although mtDNA abnormalities have been associated with metabolic syndrome and related metabolic disturbances, the specific contribution of somatic mtDNA alterations to common metabolic diseases remains incompletely defined, and their causal role, tissue specificity, and clinical significance require further investigation [55].

mtDNA copy number represents the relative abundance of mitochondrial genomes within cells and should be considered a quantitative genomic characteristic rather than a sequence variant. Changes in mtDNA copy number have been reported in obesity, T2D, cardiovascular disease, and other metabolic conditions [55,56]; however, interpretation remains challenging because measurements are strongly influenced by tissue type, cellular composition, physiological state, and analytical methodology [21,56,57]. Consequently, mtDNA copy-number changes should be interpreted cautiously and should not currently be considered equivalent to pathogenic inherited mtDNA variants or established diagnostic biomarkers for complex metabolic disease.

A further distinction is required between pathogenic and common inherited mtDNA sequence variants. The m.3243A>G variant in *MT-TL1*, classically associated with MELAS, is also a well-established cause of maternally inherited diabetes and deafness [58]. Conversely, m.16189T>C, located within the mitochondrial control region, has been associated with an increased risk of T2D and related metabolic traits in some populations; however, these associations have not been consistently replicated across populations, and its contribution to impaired glucose metabolism remains uncertain [50]. Similarly, other common mtDNA variants and haplogroup-associated variants have been investigated as potential modifiers of susceptibility to complex metabolic diseases; however, the strength and direction of these associations often vary across populations. Therefore, these findings should be interpreted cautiously, taking into account ancestry, population structure, demographic history, environmental exposures, and the mito-nuclear genetic background.

## 5. Mitochondrial Haplogroups in Human Adaptation and Disease Susceptibility

Haplogroups provide an integrative framework linking human evolutionary history with functional variability in mitochondrial biology. They are defined by characteristic combinations of maternally inherited mtDNA variants that have accumulated along different maternal lineages over time. Haplogroup analysis has therefore been widely used in population genetics to reconstruct maternal ancestry, population history, and human dispersal patterns [54,59].

Phylogenetic analyses estimate that the deepest divergences among present-day human mitochondrial lineages occurred approximately 130,000–170,000 years ago and involved lineages within macrohaplogroup L [59,60]. Macrohaplogroup L comprises the major African mitochondrial lineages and includes haplogroups L0–L6 [54,55]. Haplogroup L6 represents a relatively rare mitochondrial lineage reported at low frequencies in East African populations, particularly in Ethiopia and neighboring regions of the Arabian Peninsula, including Yemen [61,62]. Among these lineages, haplogroup L3 expanded beyond the African continent approximately 55,000–70,000 years ago [59,63], giving rise to the macrohaplogroups M and N (approximately 65,000–50,000 years ago), which subsequently spread throughout South and East Asia and parts of Oceania [54,55]. Macrohaplogroup M further diversified into haplogroups C, D, E, G, Q, and Z, whereas macrohaplogroup N contributed to the formation of haplogroups A, I, O, S, W, X, and Y, as well as macrohaplogroup R. The latter emerged approximately 58,000–59,000 years ago and dispersed across northern and eastern Eurasia and western Asia, giving rise to the haplogroups F, B, P, H, V, J, T, U, and K [61,64].

Native American populations derive much of their maternal ancestry from founding populations that migrated from Asia through Beringia during the initial peopling of the Americas. The subhaplogroups A2, B2, C1, C1b, C1c, C1d, C4c, D1, D4h3, and X2a entered the Americas approximately 13,000–20,000 years ago and subsequently spread from Alaska to Patagonia, with the exception of X2a, which remained localized in North America [19,62,65]. In addition, European-derived haplogroups, particularly H, followed by J, T, U, and V, as well as African-derived haplogroups such as L3, L2, and L1, are also present in the Americas, having arrived through transatlantic migration approximately 500 years ago during the Spanish conquest and colonial period of the Americas [65,66,67]. These phylogenetic relationships and proposed migration routes are represented separately in Figure 2 to distinguish mitochondrial lineage diversification from inferred population movements.

Mitochondrial haplogroups have also been investigated as potential modifiers of cardiometabolic phenotypes [50,51,52,53,54,55,56]. For example, haplogroup N9a has been associated with reduced susceptibility to T2D in Asian populations [71], whereas haplogroup T has been associated with obesity in an Austrian population [72]. Other associations between mitochondrial genetic variation and obesity, T2D, lipid metabolism, and cardiovascular traits have been reported in different populations [70,71,72,73]. However, many of these findings originate from candidate-haplogroup or population-specific studies that differ in ancestry composition, phenotype definitions, statistical approaches, and adjustment for population stratification. Consequently, replication has often been inconsistent, and some associations appear to be population- or ancestry-specific (Table 2).

More recent population-based and biobank studies have enabled a broader evaluation of common mtDNA variation in relation to complex phenotypes. These studies indicate that the effects of common mitochondrial genetic variation on cardiometabolic traits are generally modest and may vary according to ancestry, demographic history, environmental exposures, and analytical methodology. Overall, current evidence supports further investigation of mitochondrial haplogroups and common mtDNA variants as potential modifiers of complex phenotypes but remains insufficient to support their use as independent clinical predictors of cardiometabolic disease risk (Table 2).

## 6. Mitochondrial Diseases in the New Genomic Era

The study of MitDs has undergone a profound transformation, evolving from the pre-molecular era to the molecular and genomic eras. Early descriptions of mitochondrial myopathies relied primarily on histopathological hallmarks such as RRFs, first reported in 1963, together with biochemical evidence of metabolic dysfunction [28,73]. Although these approaches provided foundational insights into mitochondrial disease, their diagnostic utility was limited by phenotypic heterogeneity, variable tissue involvement, and the invasive nature of muscle biopsy.

The molecular era of MitDs began in 1988 with the identification of the first mtDNA PV associated with LHON [73]. Since then, advances in sequencing technologies have enabled the identification of numerous disease-causing mtDNA variants, as discussed above [28,33].

More recently, NGS technologies have substantially improved the molecular diagnosis of MitDs by enabling high-depth analysis of mtDNA variants and more accurate assessment of heteroplasmy. WES has further expanded diagnostic capabilities by enabling the identification of rare nuclear variants associated with MitDs. Although mtDNA variants may also be detected in WES data, reliable mtDNA analysis requires adequate mitochondrial sequencing coverage and bioinformatic pipelines specifically validated for mtDNA variant detection (Figure 3) [76]. A practical genomic diagnostic workflow should combine the clinical phenotype with the suspected molecular defect and the most informative tissue. Depending on the presentation, testing may include targeted or whole-mtDNA sequencing, nuclear mitochondrial-disease gene panels, WES, or WGS. Assay-specific sequencing depth and validated heteroplasmy detection limits are essential, particularly for low-level variants. Bioinformatic pipelines should also account for nuclear mitochondrial DNA segments (NUMTs), which can interfere with mtDNA variant calling. Large-scale deletions and duplications require methods validated for structural-variant detection, and mtDNA copy-number assessment should be interpreted cautiously because it is tissue- and method-dependent [21,56,57]. Long-read sequencing can improve characterization of complex rearrangements and haplotypes, but current limitations include platform-specific error profiles, analytical standardization, and the need for further clinical validation [72,73].

### Translating Mitochondrial Genomics into Precision Medicine

Applying mitochondrial genomics to precision medicine requires integrating PVs with heteroplasmy levels, tissue distribution, clinical phenotype, nuclear genetic background, and population-specific mitochondrial variation. Because heteroplasmy can vary considerably across tissues and with age, variant levels in peripheral blood may not accurately reflect those present in clinically affected tissues. Therefore, tissue selection and high-depth sequencing are crucial for detecting low levels of heteroplasmy and improving variant interpretation and genotype-phenotype correlations [19,21,22,23].

Molecular diagnosis also informs genetic counseling, reproductive decision-making, and individual risk assessment. Maternal inheritance and the mitochondrial genetic bottleneck can lead to substantial differences in heteroplasmy among offspring, limiting the ability to predict the phenotype based solely on the identified PVs [19,20]. Genetic counseling should therefore consider the specific variant, maternal family history, heteroplasmy, and tissue distribution. Reproductive options may include prenatal diagnosis and preimplantation genetic testing [2]. Integrating genomic and clinical information can further support patient stratification and individualized surveillance [19,21].

Although treatment remains largely supportive, emerging molecular approaches, including gene therapy and experimental strategies aimed at reducing the burden of mtDNA PVs, highlight the potential of precision medicine for mitochondrial disorders [73]. However, their clinical implementation remains limited by technical challenges, incomplete genotype–phenotype correlations, difficulties in variant interpretation, and unequal access to genomic testing and genetic counseling.

These challenges are particularly relevant in Latin America, where mitochondrial genomic studies remain limited and relatively few studies have systematically characterized mtDNA diversity [21]. Emerging technologies, including long-read sequencing, may improve the detection of complex mtDNA rearrangements and provide complementary approaches for heteroplasmy assessment [72,73]. However, further analytical validation is required before these approaches can be broadly implemented in clinical diagnostics. Expanding genomic inclusion will be crucial for improving variant interpretation and enabling the equitable implementation of mitochondrial precision medicine.

## 7. Conclusions and Future Perspectives

Mitochondrial genomics has substantially improved our understanding of the molecular basis and clinical heterogeneity of MitDs. Clinically established findings include mtDNA and nuclear DNA PVs underlying primary MitDs [24], the contribution of heteroplasmy and tissue distribution to disease expression, and the value of molecular testing for diagnosis and genetic counseling [19,21,22]. Advances in high-depth sequencing have improved the detection of low-level mtDNA heteroplasmy [21], while broader genomic approaches have facilitated the identification of nuclear variants associated with mitochondrial disease [57]. Long-read sequencing may further improve the detection of large-scale mtDNA rearrangements [73].

Five distinct categories of mitochondrial genomic and functional variation should be considered when interpreting disease associations: (1) inherited pathogenic mtDNA variants causing primary mitochondrial disorders; (2) common inherited mtDNA variants and haplogroups; (3) somatic mtDNA alterations acquired throughout life; (4) mtDNA copy-number changes; and (5) secondary mitochondrial dysfunction arising from metabolic, inflammatory, toxic, or ageing-related processes [18,24,55,56]. Although associations with T2D, obesity, CVD, and related metabolic traits have been reported [70,71,72,73], many findings are population-specific or inconsistent across studies and require independent replication and functional validation before they can be incorporated into clinical risk assessment. Moreover, this bidirectional interplay may establish a self-reinforcing cycle in which ageing, nutrient overload, insulin resistance, inflammation, and oxidative stress progressively impair mitochondrial function and mtDNA integrity, potentially amplifying metabolic disturbances [4,52,53].

Translating these advances into precision medicine will require integrating genomic findings with heteroplasmy levels, tissue context, clinical phenotype, nuclear genetic background, and ancestry, while clearly distinguishing established pathogenic mechanisms from associations of uncertain clinical significance [19,21,22]. This distinction is essential to avoid overinterpretation of mitochondrial variants and to determine which genomic findings are sufficiently robust to inform diagnosis, genetic counseling, risk stratification, or targeted interventions [21].

These priorities are particularly relevant for Mexico and other genetically diverse populations that remain insufficiently represented in mitochondrial genomic research. Studies of Indigenous Mexican populations illustrate the substantial mitochondrial diversity and complex demographic history that must be considered when interpreting mtDNA variation [6]. Future research should prioritize ancestry-aware analyses, the development of diverse and population-relevant reference datasets, standardized clinical and molecular phenotyping, and independent replication of genetic associations in adequately powered cohorts. Functional validation will also be essential for distinguishing causal variants from population-specific markers. Finally, equitable access to genomic testing, appropriate tissue-based diagnosis, variant interpretation, and genetic counseling should accompany technological advances to ensure that mitochondrial precision medicine benefits diverse populations rather than exacerbating existing healthcare disparities.

## Figures and Tables

**Figure 1:**
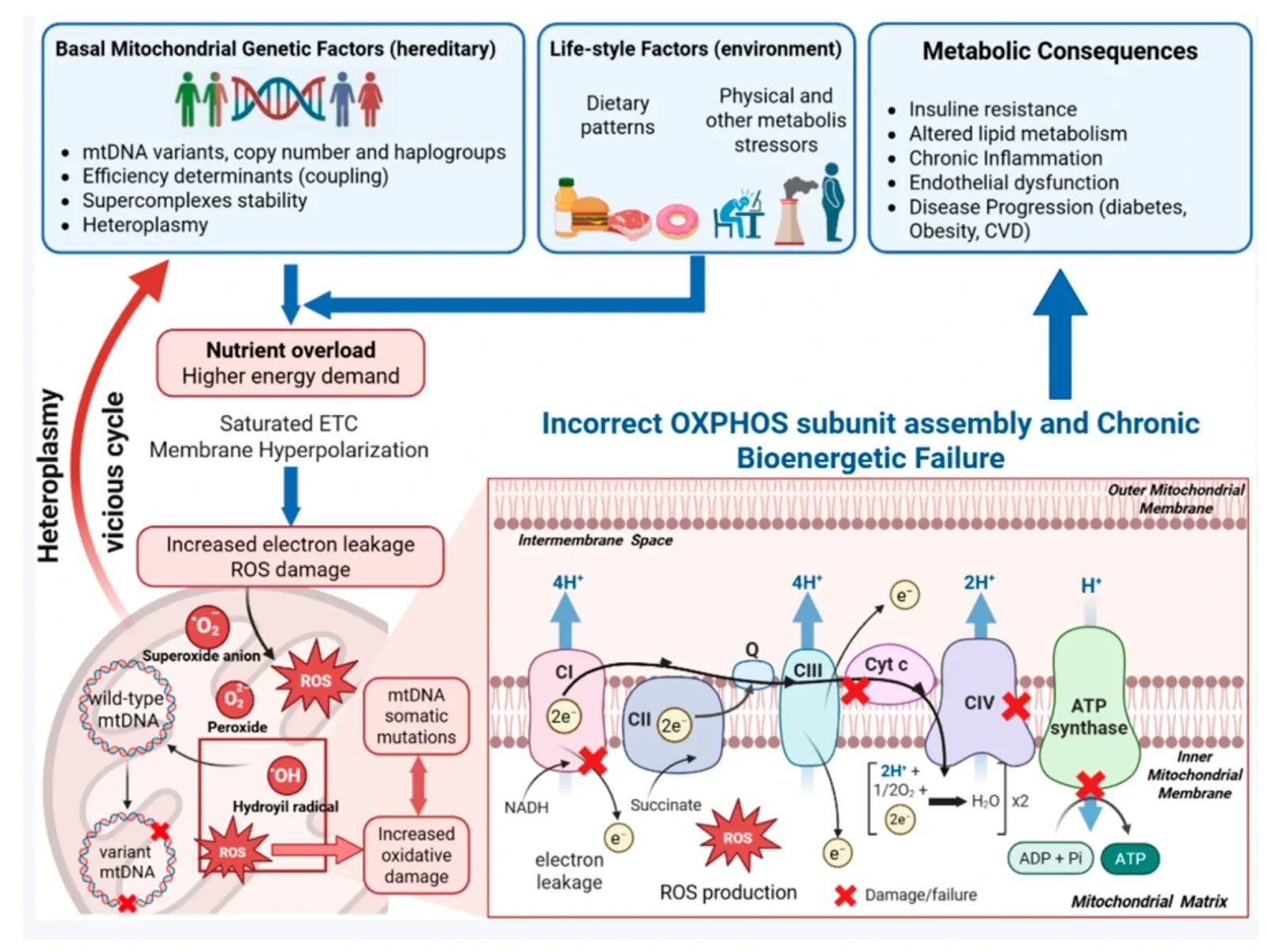
Proposed bidirectional relationship between metabolic stress and mitochondrial dysfunction. Hypercaloric diets and prolonged stress increase ROS production within the mitochondrial matrix, promoting mtDNA damage. This damage may impair the proper assembly of respiratory-chain subunits encoded by mtDNA and disrupt electron transport, resulting in electron leakage and further increases in ROS levels. In addition, impaired respiratory-chain assembly may reduce efficient proton pumping into the intermembrane space, thereby compromising oxidative phosphorylation and ATP production. ATP: adenosine triphosphate; ADP: adenosine diphosphate; Cyt c: cytochrome c; mtDNA: mitochondrial DNA; OXPHOS: oxidative phosphorylation; Pi: inorganic phosphate; Q: coenzyme Q; ROS: reactive oxygen species. Created with BioRender. Mancio, A. (2026) https://BioRender.com/tz6tjyc.

**Figure 2:**
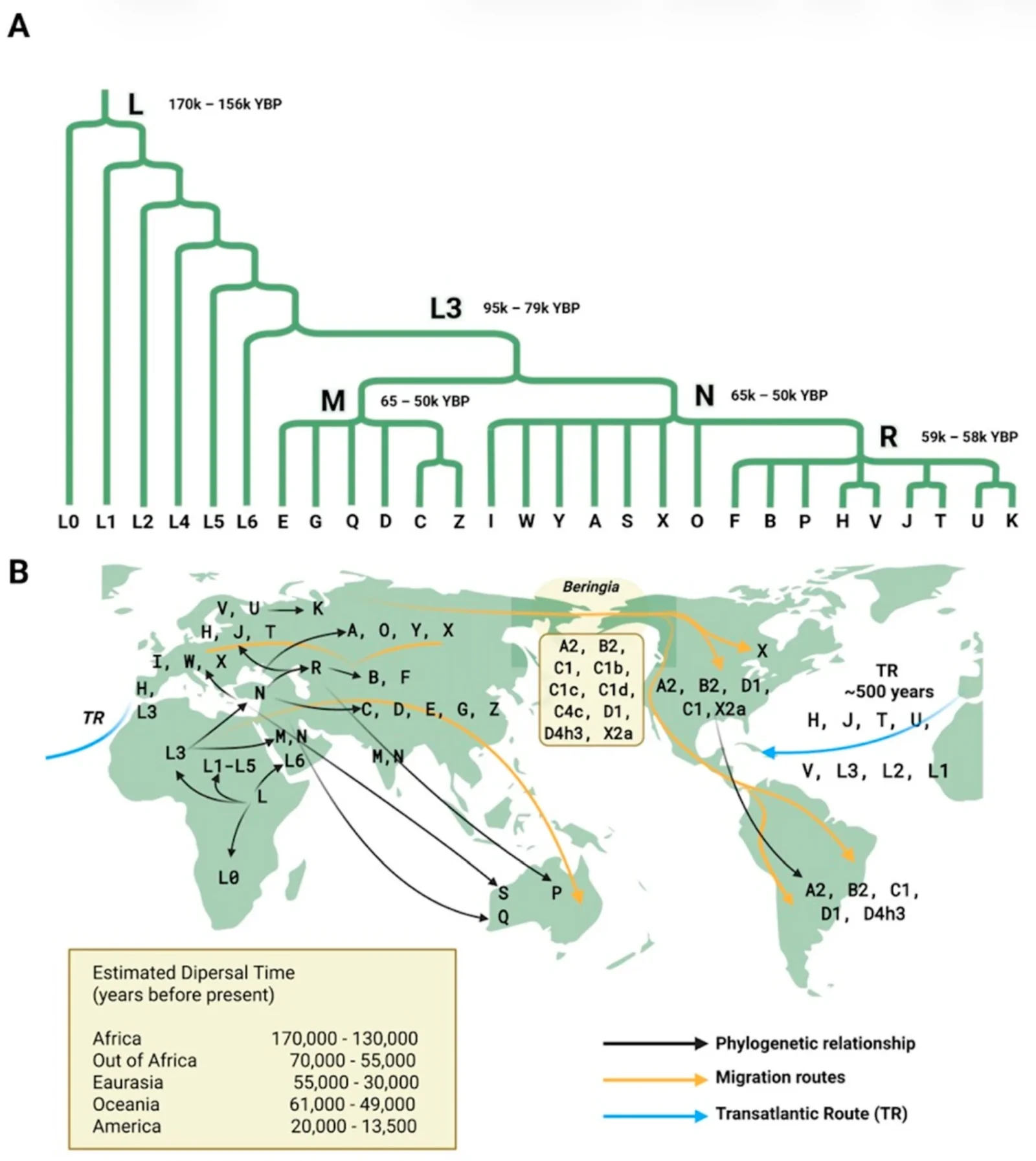
Global dispersal of human mitochondrial DNA haplogroups. (**A**) Phylogenetic relationships and estimated divergence times of major human mitochondrial DNA haplogroups [54,55,56,57]. (**B**) Migration routes. Macrohaplogroup L represents the major African mitochondrial lineages, and L3 gave rise to the major non-African macrohaplogroups M and N during the dispersal of modern humans out of Africa. These macrohaplogroups subsequently diversified across Oceania and Eurasia. Major Native American maternal lineages included A2, B2, C1, C1b, C1c, C1d, C4c, D1, D4h3, and X2a. Orange arrows indicate major migration routes during the Early, Middle, and Late Upper Pleistocene [68,69,70]; black arrows indicate phylogenetic relationships, whereas blue arrows indicate more recent migration (transatlantic routes, TR). Created with BioRender. Mancio, A. (2026) https://BioRender.com/40beidy.

**Figure 3:**
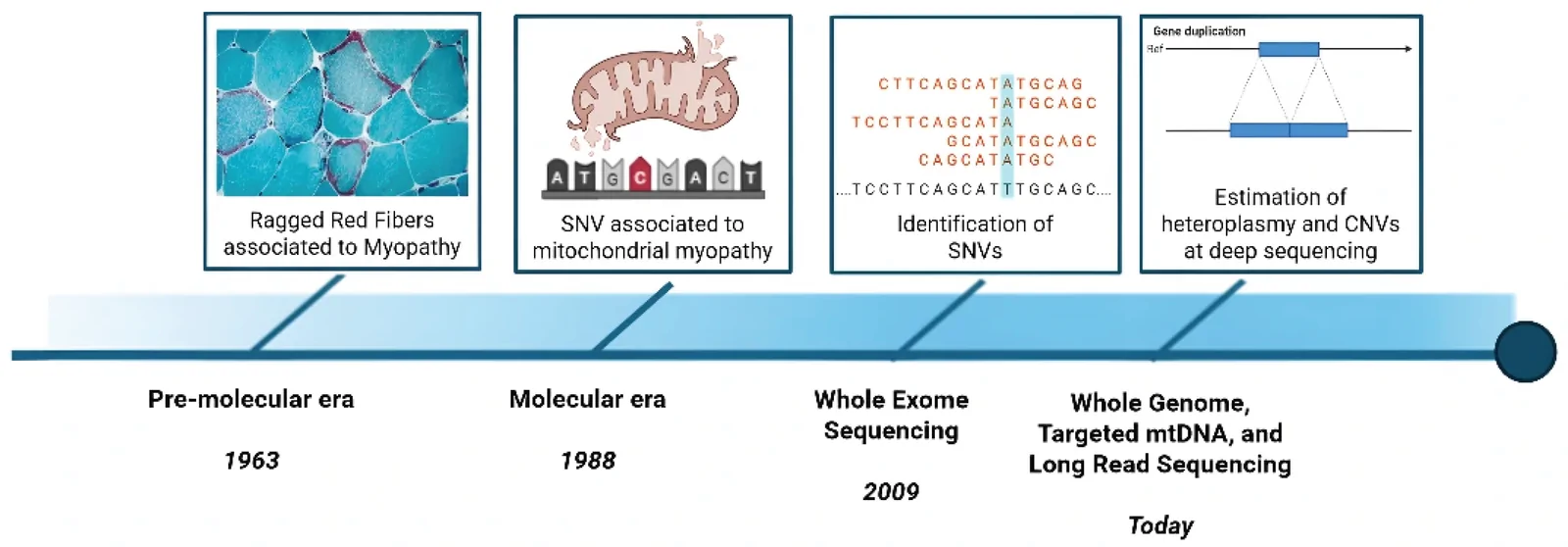
Study of mitochondrial diseases (MitDs) across the eras proposed by DiMauro. The histological image was adapted from the original article by DiMauro. SNVs: single-nucleotide variants.

**Table 1:** Comparative genetic and clinical features and diagnostic strategies for five representative mitochondrial diseases.

Disease	Major genes/Pathogenic Variants	Inheritance	Typical Heteroplasmy	Genotype-Phenotype Correlation	Preferred Diagnostic Tissue	Principal Manifestations	Molecular Testing	Therapeutics
Leber hereditary optic neuropathy (LHON)	*MT-ND4* (m.11778G>A) *MT-ND1* (m.3460G>A) *MT-ND6* (m.14484T>C)	Maternal	Usually homoplasmic; heteroplasmy has been reported in some individuals.	Incomplete penetrance. Disease expression is influenced by sex, age, mtDNA haplogroup, nuclear genetic background, and environmental factors. The likelihood of visual recovery varies according to the specific mtDNA variant.	Peripheral blood	Painless subacute central vision loss; sequential or bilateral eye involvement; impaired color vision and central scotoma; optic atrophy.	Targeted testing for the three primary variants; whole-mtDNA sequencing should be considered when the result is negative but clinical suspicion remains high.	There is currently no cure for LHON. Management includes visual rehabilitation and avoidance of mitochondrial toxins. Gene therapy remains under investigation.
Mitochondrial encephalomyopathy, lactic acidosis, and stroke-like episodes (MELAS)	*MT-TL1* (m.3243A>G, m.3271T>C)	Maternal	Usually heteroplasmic	The m.3243A>G variant is associated with marked phenotypic variability and may cause MELAS, maternally inherited diabetes and deafness, cardiomyopathy, or asymptomatic carriage. Disease severity is influenced by heteroplasmy level and tissue distribution; therefore, no reliable universal genotype–phenotype correlation has been established.	Blood initially; urinary epithelial cells; buccal cells; or muscle when blood is negative or shows low-level heteroplasmy and clinical suspicion remains high	Stroke-like episodes; seizures; encephalopathy; lactic acidosis; myopathy; hearing loss; diabetes; headache; and vomiting	Targeted testing for common pathogenic variants, followed by whole-mtDNA sequencing when targeted testing is negative but clinical suspicion remains high.	Multidisciplinary supportive management, including treatment of seizures and metabolic and cardiac complications. L-arginine and/or citrulline have been used for stroke-like episodes in some clinical settings, although evidence of efficacy remains limited.
Leigh Syndrome (LS)	*MT-ATP6* (m.8993T>G/C, m.9176T>G, m.9185T>C) *MT-ND1* (m.3697G>A) *MT-ND3* (m.10191T>C) *MT-ND5* (m.13513G>A) More than 100 nuclear genes	Maternal for mtDNA variants; autosomal recessive, X-linked or, less commonly, autosomal dominant for nuclear gene variants.	Often heteroplasmic and variant- and tissue-dependent	For MT-ATP6 variants, higher heteroplasmy levels are generally associated with more severe disease, whereas lower levels may result in milder NARP-spectrum phenotypes.	Blood initially; additional tissues depending on the suspected molecular defect and clinical phenotype.	Developmental delay or regression; hypotonia; dystonia; ataxia; brainstem dysfunction; respiratory abnormalities; lactic acidosis; and characteristic bilateral basal ganglia and/or brainstem lesions.	Complete mtDNA sequencing and nuclear gene analysis using a multigene panel, whole-exome sequencing, or whole-genome sequencing.	Multidisciplinary supportive management; treatment of respiratory, cardiac, neurological, and metabolic complications. Cofactor supplementation and a ketogenic diet may be considered.
Myoclonic epilepsy and ragged-red fibers (MERRF)	*MT-TK* (m.8344A>G; less commonly, m.8356T>C and m.8363G>A)	Maternal	Usually heteroplasmic	No clinically reliable genotype-phenotype correlation. Similar heteroplasmy levels may produce different clinical manifestations.	Blood initially; muscle or another affected tissue may increase sensitivity when blood testing is negative.	Myoclonus; generalized epilepsy; cerebellar ataxia; myopathy and muscle weakness; hearing loss; ragged-red fibers.	Molecular confirmation of PVs or whole-mtDNA sequencing may be performed.	Symptomatic multidisciplinary management, including seizure control, physical and rehabilitative therapy, hearing support, and management of cardiac and other systemic complications.
Kearns-Sayre Syndrome (KSS)	Large mtDNA deletions, most commonly the 4977-bp deletion m.8470_13446del	Usually sporadic (de novo); maternal transmission is rare.	Typically heteroplasmic	Deletion size and heteroplasmy alone do not reliably predict severity. The phenotype depends strongly on tissue distribution and the proportion of deleted mtDNA. Other single large-scale mtDNA deletions can produce a clinical spectrum that includes KSS, CPEO/CPEO-plus, and Pearson syndrome.	Skeletal muscle is particularly useful because deletions may decrease or become undetectable in the blood with age.	Progressive external ophthalmoplegia with ptosis; pigmentary retinopathy; cardiac conduction anomalities; ataxia; hearing loss; and endocrine abnormalities.	Long-range PCR and/or NGS. Specific analysis of deletions and quantification of heteroplasmy.	Supportive multidisciplinary management, including regular cardiac surveillance and treatment of conduction abnormalities, with pacemaker implantation when indicated, as well as endocrine, ophthalmological, and neurological management.

Note: Heteroplasmy values should be interpreted as variant- and tissue-specific rather than as universal diagnostic thresholds. Genotype-phenotype relationships in mitochondrial disease are influenced by tissue distribution, age, nuclear genetic background, and other biological and environmental factors. The suspected disorder, variant type, and clinical phenotype should therefore guide diagnostic tissue selection and molecular testing strategies.

**Table 2:** Representative associations between mitochondrial DNA haplogroups and cardiometabolic phenotypes across populations.

Population/Ancestry	*n*	Phenotype	Haplogroup	Effect Estimate, OR (95% CI)	*p* Value	Multiple-Testing Correction	Replication	Population-Stratification Adjustment	Reference
Kuwaiti Arabs	288	Obesity (BMI ≥ 30 kg/m^2^)	R	Protective: 0.311 (0.135–0.717)	0.006	Not reported for haplogroup analysis	No	Adjusted for age and sex	Dashti et al., 2024 [58]
			L	Risk: 2.285 (1.015–5.141)	0.046				
Southern Italian Caucasians	716	Morbid obesity	T	Risk: 1.94 (1.16–3.22)	0.004	Bonferroni correction	Confirmed by bootstrap analysis using 100 bootstrap samples	No explicit adjustment for population stratification reported	Nardelli et al., 2013 [64]
			J	Protective: 0.63 (0.45–0.89)	0.020				
Austrian Caucasians	2112	Obesity/overweight	T	Risk: 1.68 (1.2–2.3)	0.002	Bonferroni correction	Supports the association previously reported by Nardelli et al.	Adjusted for age and sex	Ebner et al., 2015 [59]
Japanese and Korean populations	4271	T2D	N9a	Protective: 0.55 (0.40–0.75)	0.0002	Not explicitly reported	Association evaluated across independent Japanese and Korean populations	Multivariable logistic regression adjusted for age and sex; no explicit adjustment for population stratification reported	Fuku et al., 2007 [60]
UK Biobank (predominantly European ancestry)	381,994	CAD, CVD, serum lipids	J, T, U	J was associated with CAD and lower total and LDL cholesterol; T and U were associated with CVD.	<0.004	Bonferroni correction; haplogroup-specific threshold *p* < 0.004	No external replication reported in study	Adjusted for age, sex, and genotyping batch; ancestry principal components were not included in haplogroup models	Cañadas-Garre et al., 2024 [74]
ALSPAC, European ancestry	7954	Cardiometabolic trajectories (BMI, lipids, BP, fat mass)	H, U, J, T, K, V, W, I, and X	No consistent evidence of association	Not applicable; results summarized using 99% Cls	99% confidence intervals	Not applicable; predominantly null findings	Sex-stratified multilevel models adjusted for relevant maternal and socioeconomic covariates	O’Neill et al., 2023 [75]

## Data Availability

No new data were created or analyzed in this study. Data sharing is not applicable to this article.

## Abbreviations

The following abbreviations are used in this manuscript:

COX	Cytochrome oxidase
CPEO	Chronic progressive external ophthalmoplegia
CSF	Cerebrospinal fluid
CVD	Cardiovascular disease
HV1	Hypervariable region 1
HV2	Hypervariable region 2
HV3	Hypervariable region 3
IMM	Inner mitochondrial membrane
IMS	Intermembrane space
LHON	Leber hereditary optic neuropathy
LS	Leigh syndrome
MELAS	Mitochondrial encephalomyopathy, lactic acidosis and stroke-like episodes
MERRF	Myoclonic epilepsy and ragged-red fibers
MitDs	Mitochondrial diseases
MM	Mitochondrial matrix
mtDNA	Mitochondrial DNA
NGS	Next-generation sequencing
OMM	Outer mitochondrial membrane
OXPHOS	Oxidative phosphorylation system
PVs	Pathogenic variants
ROS	Reactive oxygen species
RRFs	Ragged-red fibers
rRNA	Ribosomal RNA
T2D	Type 2 diabetes
tRNA	Transfer RNA
WES	Whole-exome sequencing
